# Enhanced Thermal Resistance and Mechanical Performance of Methyl Methacrylate-Based Pavement Coatings for Urban Heat Mitigation

**DOI:** 10.3390/polym17050586

**Published:** 2025-02-23

**Authors:** Kwan Kyu Kim, Yoon-Sang Choi, Hee Jun Lee, Shanelle Aira Rodrigazo, Jaeheum Yeon

**Affiliations:** 1 Korea Conformity Laboratories, Chuncheon 24341, Republic of Korea; kim89@kcl.re.kr; 2 Korea Conformity Laboratories, Hoengseong 25234, Republic of Korea; yschoi@kcl.re.kr; 3 California Department of Transportation, Fresno, CA 93728, USA; heejun.lee@dot.ca.gov; 4 Department of Regional Infrastructure Engineering, Kangwon National University, Chuncheon 24341, Republic of Korea; rodrigazo.shanelleaira@kangwon.ac.kr

**Keywords:** urban heat island (UHI), thermal insulation, pavement coating, MMA

## Abstract

The urban heat island effect raises road surface temperatures, increasing energy demands and accelerating pavement deterioration. This study evaluates a polymer-based pavement system using methyl methacrylate (MMA) resin with aluminum silicate (AS), glass bubbles (GBs), and microencapsulated n-docosane phase-change material (PCM) to identify the most effective solution. Indoor laboratory tests determined AS as the optimal choice, balancing thermal insulation, workability, and mechanical strength. AS-containing mixtures reduced surface temperatures by ~10 °C and exhibited superior compressive strength (28.2 MPa at 6 wt%) compared to GB (23.7 MPa at 4 wt%) and PCM (27.2 MPa at 6 wt%). AS also maintained stable viscosity at ≤10 wt%, unlike GB and PCM, which became unworkable above 5 wt%. The AS-based system achieved high skid resistance (90.2 BPN), abrasion resistance (0.1% wear after 500,000 cycles), and low VOC emissions (69.64 g/L). Adjusting the resin-to-BPO ratio to 1:0.42 enabled a 30 min curing time at 25 °C, ensuring practical application. These findings highlight AS as the most effective filler for large-scale deployment. Future work should assess long-term durability and optimize formulations for broader adoption in heat-mitigating infrastructure.

## 1. Introduction

Elevated surface temperatures in urban environments, commonly referred to as the urban heat island (UHI) effect, have been shown to exacerbate cooling energy demands, accelerate infrastructure degradation, and pose significant public health risks. Prior research indicates that the conversion of vegetated areas into impervious surfaces substantially amplifies UHI effects by trapping heat and diminishing natural cooling mechanisms [1,2,3]. While Wu and Liu [4] demonstrated that UHI conditions can influence building energy consumption—reducing heating loads by 15.8% but increasing cooling demands by up to 30%—their study did not examine the efficacy of advanced pavement coatings as a comprehensive mitigation strategy. Additional investigations, including those conducted in Sri Lanka and parts of China, highlight the accelerated deterioration of pavement materials under prolonged thermal exposure and the heightened risks posed to vulnerable populations [1,5,6]. However, these analyses have mainly focused on macro-scale urban planning and green-space integration, without addressing the potential of specialized thermal insulating coatings for road surfaces.

Initial strategies for mitigating road surface temperatures have primarily relied on the application of reflective pavements and high-albedo materials. For example, one study reported average temperature reductions of 2–4% in a high-reflectance campus setting [2], yet it did not account for mechanical wear or real-world traffic loads. Similarly, research exploring the incorporation of ceramic-waste aggregates and hollow fillers in asphalt has demonstrated temperature reductions of up to 11.5 °C, though such approaches have exhibited trade-offs in terms of rutting resistance and low-temperature cracking [7,8]. More recent advancements involving vacuum insulation and thermoelectric additives suggest additional potential benefits; however, these studies have largely been limited to short-term testing and experimental prototypes that lack scalability [9,10]. In many cases, existing treatments have failed to provide comprehensive validation of their mechanical performance or to integrate multiple critical functionalities, including heat mitigation, durability, cost-effectiveness, and skid resistance.

To address these limitations, recent research has focused on high-reflectivity surface coatings with improved thermal insulation. Some coatings have achieved considerable temperature reductions (e.g., up to 10 °C) while also exhibiting superior bond strength and abrasion resistance compared to conventional materials [11,12]. However, these improvements have not consistently been validated over extended service periods or under continuous vehicular loads, raising questions regarding their economic viability and long-term durability. Photocatalytic and superhydrophobic coatings, for instance, provide additional environmental advantages, such as the reduction of nitrogen oxide (NOx) emissions and self-cleaning properties [13,14]; yet, challenges related to large-scale implementation and potential degradation over time remain unresolved.

Comparative analyses of thermal insulating paints and conventional asphalt concrete underscore the substantial advantages that advanced coatings can deliver. Certain nanocomposite formulations incorporating silica aerogels, titanium dioxide, or barium sulfate (BaSO_4_) have demonstrated significantly lower thermal conductivity and reflectance values exceeding 90% [15,16,17]. These coatings not only mitigate pavement surface temperatures more effectively than traditional asphalt but also exhibit promising mechanical properties. For example, the inclusion of mesoporous silica and engineered micro- and nanotextured morphologies enhances wear resistance and adhesion [15,18,19]. Additionally, investigations into UV-curable and polymer-based coatings highlight solvent-free production, rapid application, and reduced volatile organic compound (VOC) emissions, suggesting a favorable life-cycle cost–benefit ratio relative to asphalt’s higher long-term maintenance demands [20,21,22,23]. Economic analyses further indicate short payback periods—less than six months—due to resulting energy savings and decreased heat-related maintenance expenses [24].

Despite these promising findings, several critical research gaps persist. Many studies have focused on building envelopes or controlled laboratory environments, providing valuable insights into thermal reflectivity but failing to assess the real-world performance of these coatings on high-traffic roads. Another limitation concerns the integration of PCMs, which have the potential to moderate peak thermal loads by absorbing and releasing latent heat. However, relatively few pavement studies have systematically evaluated the combined mechanical and thermal effects of PCMs [25,26]. Similarly, while filler aggregates such as AS and GB have been investigated for their potential to improve thermal insulation and mechanical properties, their individual contributions remain insufficiently explored in field applications. Additionally, while certain studies emphasize the importance of durability, chemical stability, and crack resistance in coatings designed to withstand intense mechanical stress, few studies present multi-year field data or comprehensive cost analyses [27,28,29].

To address these gaps, this study critically evaluates thermal insulating paints in comparison to conventional asphalt concrete in terms of performance and durability. First, it explores how novel coating technologies achieve substantial temperature reductions under high solar loads. Then, it examines the extent to which these coatings retain or enhance mechanical properties such as wear resistance and adhesion, ensuring resilience in real-world traffic conditions. To facilitate a comprehensive comparison, this study proposes a structured evaluation framework to assess temperature reduction under both indoor and outdoor conditions, while verifying that the materials conform to established standards. This analysis aims to elucidate the tangible benefits of advanced surface technologies for mitigating urban heat stress.

Ultimately, by synthesizing findings from a diverse range of studies and product trials, this discussion seeks to inform both pavement engineers and urban planners regarding the technical viability of next-generation thermal insulating paints. Furthermore, it highlights the necessity of multi-functional approaches that integrate thermal reflection, insulation, and mechanical stability. These insights can help guide the development of large-scale field trials and inform policy decisions, supporting urban adaptation strategies to mitigate the adverse effects of the UHI phenomenon on energy consumption, infrastructure longevity, and public health.

## 2. Materials and Methods

### 2.1. Materials

**Figure 1:**
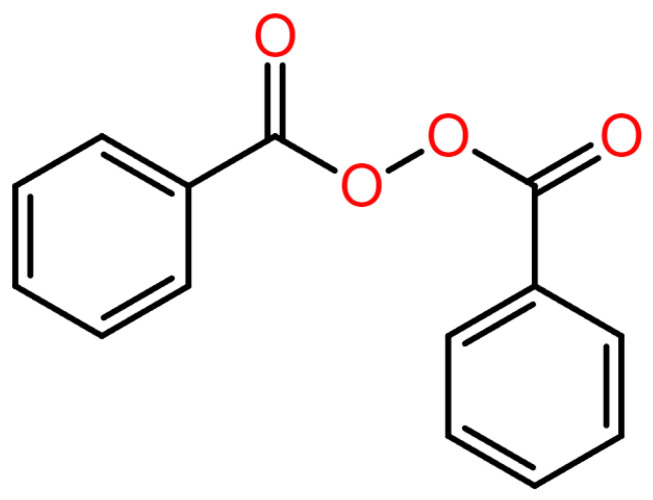
Molecular structure of Benzoyl Peroxide (BPO).

Methyl methacrylate (MMA) resin was chosen for its excellent chemical resistance, abrasion resistance, and weathering properties [20]. In particular, its ability to withstand UV exposure and maintain mechanical integrity makes it suitable for high-temperature environments. The key physical properties of the MMA resin are presented in Table 1. Benzoyl Peroxide (BPO), whose properties are shown in Table 2 and structure in Figure 1, was used as the curing agent to initiate polymerization of the MMA resin and achieve a rapid yet controllable setting time. Both materials were sourced from Samkientech Co., Ltd., Yongin, Republic of Korea.

Aluminum silicate (AS) (SDB Corp., Ansan, Republic of Korea), also referred to as magnesium aluminum silicate, was selected for its evident effect on thermal reflectivity. During mixing and curing, AS particles help form closed air layers that reduce heat conduction within the pavement matrix. The version employed here is a hollow, spherical powder with a pH range of 6–8. More specific physical parameters, such as particle size and thermal conductivity, are displayed in Table 3.

Glass bubbles (GBs) (3M™ VS5500, Seoul, Republic of Korea), composed primarily of soda–lime–borosilicate glass, were incorporated to further enhance the insulating capacity of the pavement. These hollow, lightweight spheres also provide a “ball-bearing” effect that preserves flowability during mixing, even when introduced at higher proportions [30]. The specific brand of GB in this research featured a density range of 0.30–0.39 g/cc. Other relevant material properties are summarized in Table 4.

PCM was integrated into the resin to regulate surface temperatures through latent heat absorption during phase transitions. Initial attempts using raw n-octadecane PCM (PARAFOL 18-97, Sasol Chemicals (USA) LLC, Houston, TX, USA) were unsuccessful due to premature solidification during mixing (at stirring speeds below 150 rpm), uneven dispersion, and incompatibility with the hydrophobic MMA resin. By activating at 44 °C, the encapsulated PCM effectively mitigates temperature spikes within the most critical range for hot-weather pavement stress. Stirring and curing times are discussed in detail in the Results Section (Section 3.1). Its inclusion in the pavement formulation is anticipated to mitigate heat-induced damage, extend pavement lifespan, and enhance serviceability during summer conditions. Key properties of n-docosane (PARAFOL 22-95, Sasol Chemicals (USA) LLC Houston, TX, USA) are summarized in Table 5.

To meet the manufacturer’s specification, No. 3 silica sand (~1.2 mm grains size) was utilized as the aggregate to enhance the mechanical properties of the pavement. This filler facilitates effective load distribution and improves surface roughness, thereby minimizing skidding under both wet and dry conditions. The silica sand content was maintained at approximately 20% by weight relative to the resin, ensuring an optimal balance between mechanical stability and mix workability. Its inclusion contributes to the structural integrity and load-bearing capacity of the pavement, enhancing durability under diverse traffic and environmental conditions.

**Figure 2:**
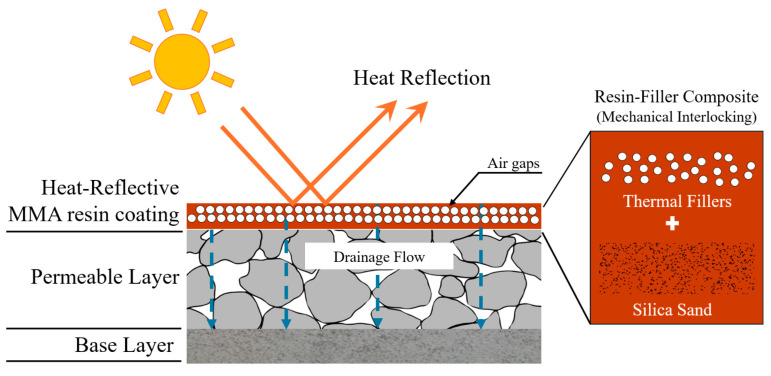
Schematic diagram of the heat-reflective MMA resin pavement system.

Figure 2 presents a schematic overview of the heat-reflective MMA resin pavement system. In this configuration, thermal fillers—namely aluminum silicate (AS), glass bubbles (GB), and phase-change material (PCM)—are dispersed within the MMA resin matrix to enhance thermal performance. The permeable layer beneath facilitates drainage, while the silica sand aggregate ensures mechanical interlocking and load-bearing capacity. Through a combination of solar reflectivity and latent heat absorption, this system aims to mitigate surface temperature spikes under high-temperature conditions.

### 2.2. Design and Preparation of Resin

The resin formulation was devised to achieve an optimal synergy between thermal insulation and mechanical robustness. Mixing operations were conducted at 25 ± 2 °C. The MMA resin was combined with BPO at a ratio of 1:0.42, which extended the curing time to approximately 30 min—a timeframe deemed practical for on-site applications, accounting for operational requirements.

In preliminary experiments, the concentrations of AS, GB, and PCM were systematically increased from 0% to 10% in increments of 2–5%, allowing for an evaluation of their effects on flowability, curing time, and overall workability. Threshold levels were determined when the mixture’s viscosity increased to the point that stirring at 150 rpm became impractical. These observations informed the final formulation, ensuring that the resin maintained both mechanical robustness and thermal efficiency for practical field applications (see Section 3.1 for a detailed discussion).

**Figure 3:**
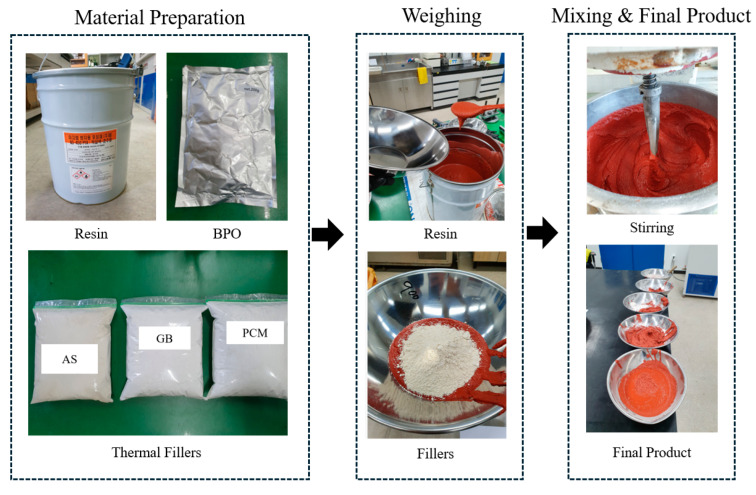
Overview of the manufacturing process.

After these preliminary tests, the manufacturing proceeded as shown in Figure 3. The first step, material preparation, involves assembling the essential raw materials (resin, BPO, and thermal fillers such as AS, GB, and PCM). In the weighing phase, each component is measured precisely to maintain accurate proportions and ensure consistency in the final formulation. To further enhance both mechanical and thermal properties, an inorganic red pigment was incorporated. This measure was introduced to improve road visibility, with the distinct red hue serving as a visual cue for drivers, enhancing traffic safety by aiding in accident prevention and increasing overall road awareness.

**Figure 4:**
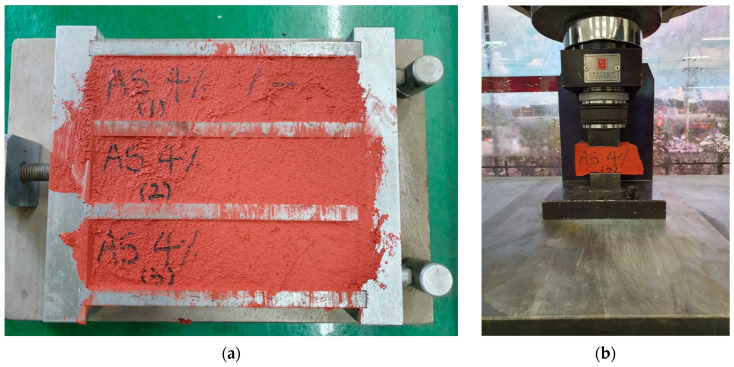
Sample test specimen: (**a**) Photograph of the MMA-based coating formulation prepared with 4% aluminum silicate. (**b**) A representative compressive test specimen used for mechanical evaluation.

Following the mixing process, the products were then allowed to dry and tested for compressive strength using prismatic specimens measuring 40 × 40 × 160 mm, with a loading rate of 800 N per second (Figure 4). These tests were performed in accordance with the performance standards of ASTM C349 [31], ensuring compliance with established benchmarks. The compressive strength testing was conducted using specimens with varying proportions of AS, GB, and PCM (0%, 2%, 4%, 6%, 8%, and 10%) to evaluate the impact of additive content on mechanical performance.

### 2.3. Testing Methods

#### 2.3.1. Indoor Thermal Resistance Testing

Indoor testing focused on evaluating the thermal and mechanical properties of the pavement materials under controlled laboratory conditions. Due to the lack of established standards for testing heat resistance in pavement materials, this study employed experimental test methods derived from existing research on heat-resistant performance [8,11,32]. The indoor heat resistance test was conducted within a purpose-built chamber measuring 180 cm in height, 50 cm in width, and 50 cm in depth. Insulation materials (50 mm thick) were applied to all interior surfaces to minimize external environmental effects on the specimen temperatures. Each test sample was placed in a dedicated compartment within the chamber to prevent thermal interference between specimens.

**Figure 5:**
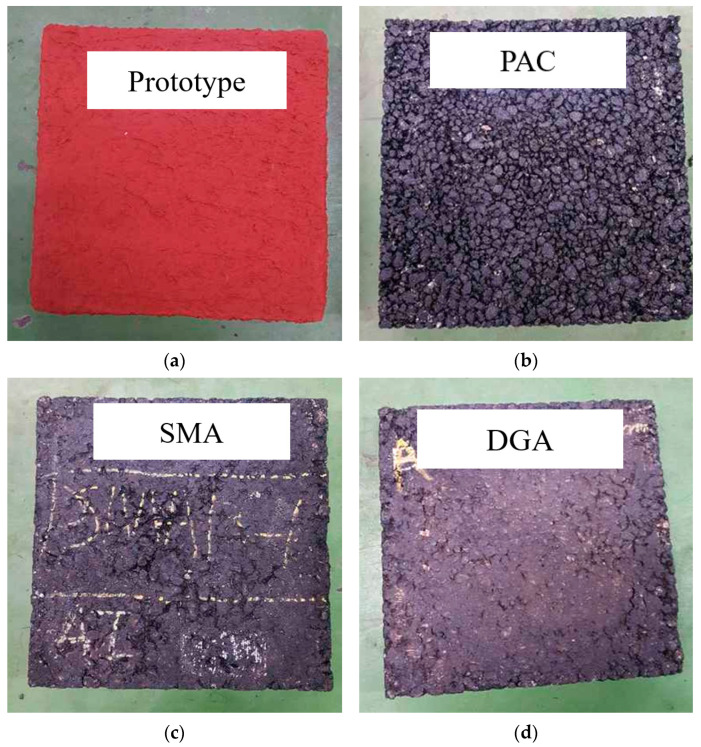
Test specimens; (**a**) developed product; (**b**) porous asphalt concrete (PAC); (**c**) Stone mastic asphalt (SMA) concrete; (**d**) dense-graded asphalt (DGA) concrete.

To facilitate comprehensive analysis, a single batch of specimens was prepared (Figure 5), consisting of the developed product (Prototype) and three comparative specimens representing common asphalt types: porous asphalt concrete (PAC), stone mastic asphalt (SMA), and dense-graded asphalt concrete (DGA). The same set was used for both indoor and outdoor evaluations.

**Figure 6:**
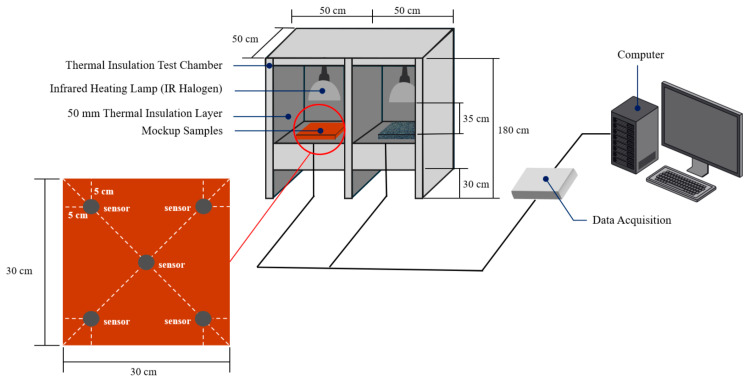
Schematic diagram of the heat resistance test setup.

Temperature data were collected using a Lutron TM-947SD thermoelectric recorder (Lutron Co., Ltd., Seongnam, Republic of Korea). Specifications of the heat resistance test equipment are summarized in Table 6, and the schematic diagram of the test setup, illustrating the arrangement of halogen lamps, test samples, and temperature sensors, is presented in Figure 6. A 650 W halogen lamp served as the primary heat source. Although the initial apparatus design positioned the lamp 100 cm away from the samples, preliminary trials revealed only negligible temperature increases even after two hours of continuous heating. Consequently, the lamp-to-specimen distance was reduced to 35 cm to achieve a more appreciable temperature elevation within the two-hour test window.

**Figure 7:**
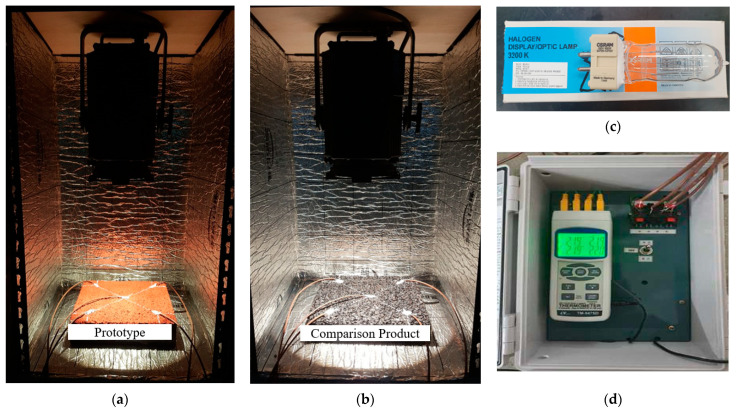
Heat resistance test components: (**a**) developed sample placed in the test chamber; (**b**) control sample placed in the test chamber; (**c**) halogen lamp; (**d**) lamp control unit and temperature data acquisition devices.

Before each test, specimens were conditioned at room temperature (20 ± 5 °C) for at least 12 h. Five temperature sensors were affixed to each specimen, as shown in Figure 6, to capture spatial variations in temperature. These sensors were connected to a temperature data acquisition system. Once the lamp was activated, data were logged every 10 min for the first hour and every 30 min thereafter, up to a total duration of two hours. At the conclusion of the two-hour period, the final temperatures of the specimens were recorded and compared against those of a control (e.g., unmodified asphalt concrete) to quantify any improvement in heat-resistant performance. Figure 7 provides a detailed breakdown of the components involved in the heat resistance test: Figure 7a shows the developed test sample placed in the test chamber, Figure 7b depicts the control test sample within the chamber, Figure 7c features the halogen lamps used as the heat source, and Figure 7d presents the lamp control unit along with the temperature data acquisition devices. Once the heat source was activated, temperature data were continuously recorded every 10 min for the first hour, followed by 30 min intervals up to a total duration of two hours.

#### 2.3.2. Outdoor Thermal Resistance Testing

Field testing comprised two separate tests designed to verify the results obtained from laboratory experiments and to demonstrate the real-world applicability of the heat-reflective pavement materials. Since the project timeline did not align with the peak summer season, direct sunlight alone was insufficient for clearly observing thermal differences. Consequently, in the first test, an artificial heat source was used outdoors in a setup analogous to the indoor tests, while the second test involved the actual application of the pavement mixture on a bicycle path for extended skid resistance testing.

In the absence of intense natural sunlight, the heat resistance test procedure from the laboratory was replicated outdoors. The same 650 W halogen lamp was employed to provide a controlled heat input, ensuring consistent and comparable thermal loading for both the heat-reflective pavement samples and the unmodified asphalt concretes. The lamp was positioned at a fixed distance from the samples—mirroring the indoor protocol—to compensate for lower ambient temperatures.

**Figure 8:**
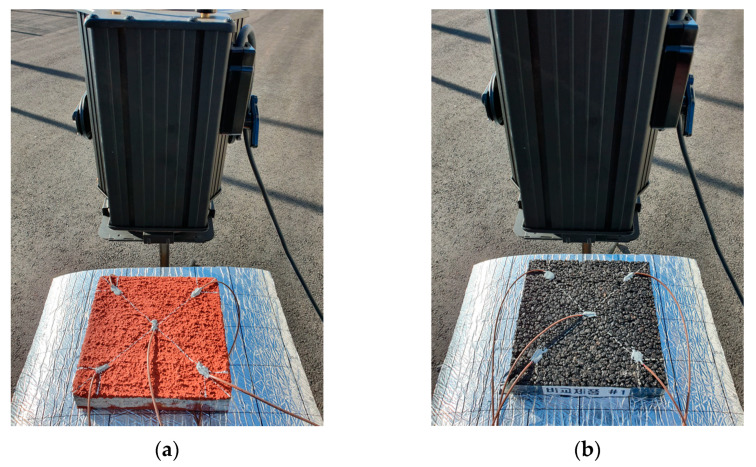
Outdoor test setup: (**a**) developed sample; (**b**) comparison product.

As in the indoor tests, surface temperatures were recorded at 10 min intervals during the first hour and every 30 min thereafter until a total testing time of two hours elapsed. Multiple thermocouple sensors were attached at equal intervals on each specimen’s surface to capture any localized temperature variations. By performing these measurements in an open-air environment, this study was able to observe whether external factors, such as slight wind or fluctuating ambient temperature, affected the heat-reflective performance in ways that would not be evident indoors. Figure 8 illustrates this outdoor test setup.

#### 2.3.3. Mechanical Performance Evaluation

Mechanical performance was evaluated both in the laboratory and in the field to verify that the incorporation of heat-resistant additives did not compromise pavement structural integrity or safety.

**Laboratory Mechanical Testing.** In the laboratory, the pavement mixture (excluding primer or resin) underwent a comprehensive series of mechanical tests to evaluate its performance and durability. Volatile organic compound (VOC) emissions were quantified in accordance with ASTM D3960 standard [33]. Under this standard, the VOC content for primers was restricted to a maximum of 400 g/L to ensure environmental compliance.

Mechanical properties were assessed following EN 13197 [34], encompassing both adhesion strength and abrasion resistance. Adhesion strength was determined using the equation σt=PA, where $\sigma_{t}$ represents adhesion strength (MPa), P is the maximum applied load (N), and A is the minimum cross-sectional area of the specimen (mm^2^). The resulting adhesion strength values were evaluated against a performance threshold of ≥1.5 MPa to ensure adequate bonding characteristics.

Abrasion resistance was measured using the wear rate formula:

(1) $$Wear\;Rate(\%)=\frac{Initial\;Thickness-Final\;Thickness}{Number\;of\;Wheel\;Passages}\times 100$$

This assessment, conducted in accordance with EN 13197, ensured that the pavement material maintained its structural integrity under repeated mechanical stress, thereby verifying its resistance to wear and tear. Additionally, thermal stability was evaluated to determine the pavement’s resilience in low-temperature conditions. The material was subjected to a −10 °C requirement, assessing its ability to withstand extreme cold without compromising its mechanical properties. These laboratory evaluations collectively confirmed the pavement mixture’s suitability for long-term application in demanding environments, demonstrating its compliance with relevant standards and its capacity to perform reliably under various operational conditions.

**Field Mechanical Testing.** To further confirm the practicality and durability of the heat-reflective pavement, a full-scale application was conducted on a bicycle path at Gongchoncheon Sports Park in Incheon, Republic of Korea. Prior to installation, the existing surface was swept and flushed to remove any debris. A primer coat was then applied, followed by the newly formulated pavement mixture containing the AS–based binder. This mixture was distributed evenly using a roller to achieve consistent thickness and integrate the heat-reflective additives throughout the surface.

**Figure 9:**
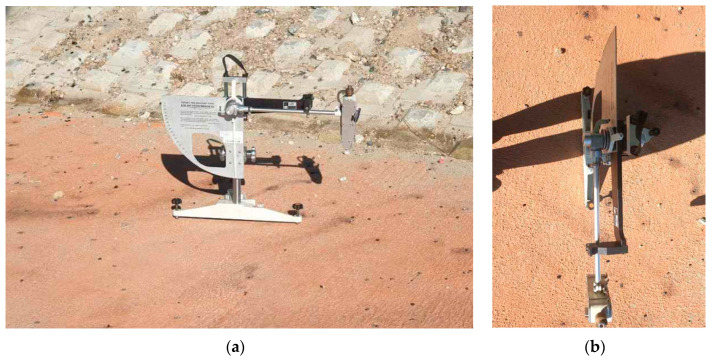
BPT setup for skid resistance evaluation: (**a**) front view of BPT; (**b**) top view of BPT.

After on-site construction, the mechanical performance of the pavement was evaluated through a skid resistance test conducted using the British Pendulum Tester (BPT), as illustrated in Figure 9. The test was performed under actual operating conditions in accordance with EN1824 [35], a standard that defines the requirements for load-bearing capacity, abrasion resistance, and durability. The results of the BPT confirmed that the heat-reflective pavement system not only provided thermal benefits but also maintained essential safety standards, including adequate skid resistance, thereby demonstrating its suitability for practical applications.

## 3. Results and Discussion

### 3.1. Pavement System

**Figure 10:**
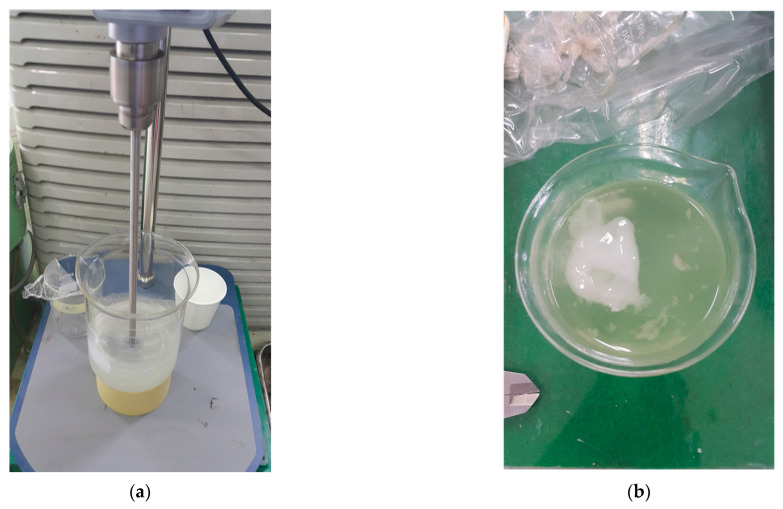
Influence of excessive filler loading on stirring efficiency and mixture homogeneity; (**a**) mechanical stirring of the resin–filler mixture; (**b**) representative example of partial solidification.

After replacing n-octadecane with n-docosane PCM, the formulation achieved stable dispersion, effectively preventing phase separation during mixing. The encapsulated n-docosane enhanced thermal regulation by absorbing and releasing latent heat at 44 °C, ensuring effective heat mitigation under real-world pavement conditions. However, challenges arose during the mixing process when the additive content was not optimized. Increasing the AS content beyond 10% significantly impeded stirring at mixing speeds below 150 rpm (Figure 10), reducing the overall workability of the mixture. This issue was further exacerbated by the mixture’s tendency to solidify, making stirring increasingly difficult and ultimately rendering the formulation ineffective. Similarly, for GB and PCM, stirring became unmanageable when their concentrations exceeded 5%, underscoring the critical need to optimize additive content to maintain a balance between material performance and processability.

To further optimize the curing period without compromising performance, the resin-to-BPO ratio was adjusted from 1:0.6 to 1:0.42, resulting in a curing time of approximately 30 min at 25 °C. Maintaining a constant resin-to-hardener ratio ensured that the curing kinetics were governed by the MMA resin–BPO reaction. Importantly, because the fillers (AS, GB, and PCM) are inert with respect to free-radical polymerization, even in high proportions, they did not affect the fundamental reaction rate of the MMA resin–BPO system [20,26]. Any slight variations in curing time (Table 7) may be attributed to minor measurement inconsistencies rather than an intrinsic effect of the fillers. This confirms that while each filler can enhance thermal insulation and mechanical strength, they do not significantly impact the curing kinetics of the resin–BPO system.

**Figure 11:**
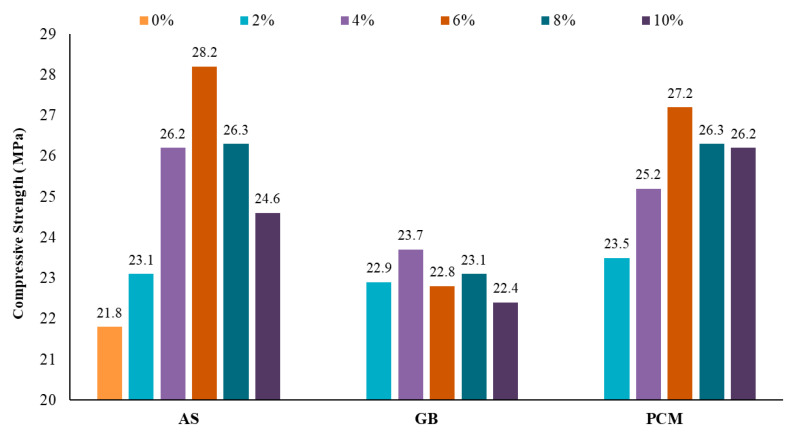
Results for compressive strength at different proportions of AS, GB, and PCM.

In terms of mechanical performance, Figure 11 shows that the AS-containing formulation reached a maximum compressive strength of 28.2 MPa at 6 wt%, while the PCM-containing formulation achieved 27.2 MPa at the same loading, and the GB-containing formulation peaked at 23.7 MPa at 4 wt%. Considering that PCM is generally more expensive and that AS can be incorporated at higher loadings without compromising stirring efficiency, the evaluations indicate that AS provides the best overall balance of mechanical performance, workability, and cost-effectiveness. Consequently, although GB and PCM were assessed as potential thermal fillers, AS was ultimately selected as the final filler for the pavement system formulation. Based on these findings, a loading range of 1–10 wt% for AS is recommended to optimize performance and maintain practical workability during mixing.

### 3.2. Thermal Performance Results

**Figure 12:**
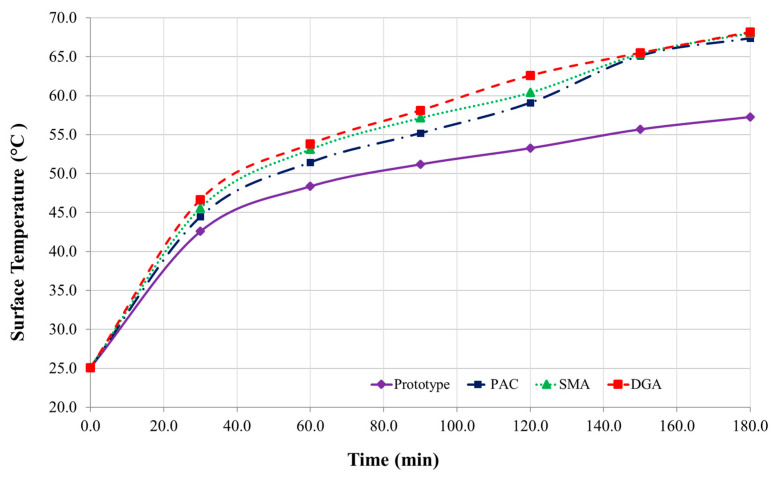
Indoor thermal performance results across specimens.

**Figure 13:**
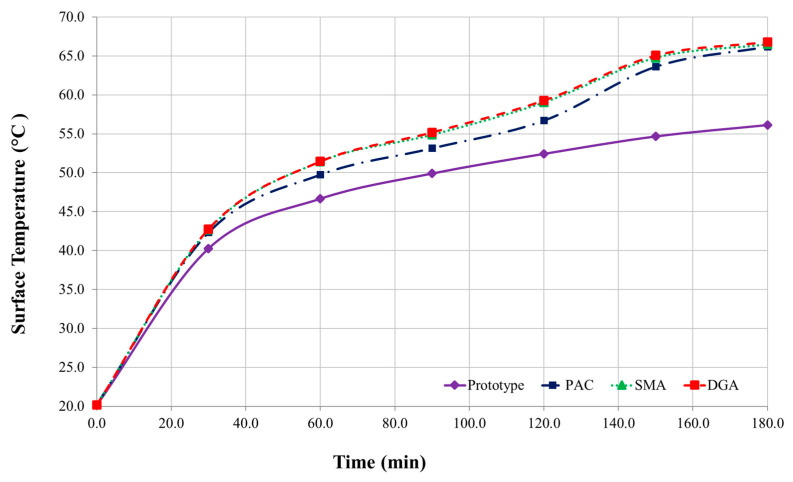
Outdoor thermal performance results across specimens.

The indoor and outdoor thermal resistance tests confirm that the newly formulated pavement system consistently achieves surface temperature reductions of approximately 10 °C compared to conventional asphalt concretes. Initially, temperature differences between the prototype and reference specimens are minimal. However, as testing progresses, these differences increase steadily. By the 180 min mark, the prototype reached a surface temperature of 10.1–10.7 °C lower in outdoor trials and 10.08–10.90 °C lower in indoor tests (Figure 12 and Figure 13). This performance may be attributed to the incorporation of AS particles and GB, which create air pockets that significantly impede conductive heat transfer within the pavement’s polymeric matrix.

Both indoor and outdoor experiments exhibit similar trends in temperature reduction, with minor fluctuations in outdoor measurements due to wind and ambient conditions. The prototype remains consistently cooler than the reference asphalts, reinforcing its potential to mitigate urban heat island effects and reduce associated energy demands. A distinct thermal pattern emerges: within the first 30 min, temperature differences increase rapidly, followed by a more gradual rise until approximately 150 min, after which they stabilize, maintaining reductions of over 9 °C across all comparisons (Table 8).

This sustained thermal performance is particularly advantageous in urban environments where pavements are continuously exposed to vehicle loads, daily temperature changes, and unpredictable weather. The consistent cooling effect throughout the tests highlights the reliability of the design, making it a strong candidate for reducing energy use and improving comfort in city spaces. While this study focused on a 180 min period, future research could extend the observation time, explore how seasons, humidity, and airflow impact performance, test the pavement under extreme temperatures, and further evaluate its long-term energy-saving potential.

### 3.3. Material Validation and Field Implementation

Beyond thermal considerations, the newly developed pavement system exhibits mechanical characteristics that are conducive to full-scale deployment in traffic-intensive areas, as shown in Table 9. Its drying time is approximately 40 min. Laboratory data further show that the material meets or surpasses relevant structural criteria, with adhesion strength reaching 2.12 MPa, exceeding the ≥1.5 MPa benchmark, coupled with a measured VOC content of 69.64 g/L—far below the 400 g/L regulatory threshold. These findings indicate minimal disruption during installation and compliance with standard health protocols. Field validation at Gongchoncheon Sports Park further demonstrated the system’s traffic safety performance, with skid resistance measurements of 90.2 BPN, exceeding the 55 BPN target, and abrasion resistance emphasizes the coating’s resilience under repeated loading with only 0.1% wear. These results indicate that the developed coating not only enhances heat mitigation but also ensures long-term mechanical resilience, making it a strong candidate for large-scale deployment in high-traffic urban environments.

### 3.4. Integration of Thermal and Mechanical Properties

Traditional heat-resistant coatings often prioritize thermal reflectivity at the expense of mechanical durability, leading to issues such as poor adhesion and premature wear [36]. In contrast, the developed polymer-based pavement system was evaluated with different thermal fillers to achieve a comprehensive performance profile. The MMA resin serves as a strong polymer matrix, providing chemical resistance and structural integrity. Assessments reveal that hollow AS particles enhanced thermal insulation through the creation of insulating air gaps, GBs provided additional thermal barriers and improved flowability [15], and PCM facilitated latent heat absorption during phase transitions [26]. The “ball-bearing” effect of GBs further contributed to easier mixing and application, thereby enhancing overall efficiency. When PCM was included, it stabilized surface temperatures and mitigated thermal spikes, corroborating previous studies indicating that PCM-integrated pavements may reduce surface temperatures and enhance user comfort [25].

Mechanically, the pavement system demonstrated high compressive strength, excellent abrasion resistance, and sustained skid resistance under dynamic loading conditions [11]. Its resistance to environmental factors, such as salt exposure, further contributes to durability. Moreover, the material’s thermal-resistant properties render it suitable for various urban applications beyond pavements, including rooftops, building facades, parking areas, and school playgrounds. Emerging sustainable pavement strategies, such as incorporating agricultural waste fibers into asphalt binders to enhance rutting and fatigue resistance [37], underscore a broader trend towards multi-functional, eco-efficient infrastructure solutions. Although these approaches were not combined in a single formulation here, each offers complementary benefits that could inform future low-carbon pavement designs addressing both mechanical robustness and urban heat island mitigation.

By evaluating and integrating the distinct thermal and mechanical benefits provided by individual fillers, the polymer-based pavement system offers a viable solution for urban heat challenges without compromising essential performance metrics. The consistent results across both controlled laboratory environments and practical field applications affirm the material’s readiness for large-scale adoption, presenting a significant advancement in polymer-based infrastructure materials.

## 4. Conclusions

This study developed a heat-reflective pavement system that reduces urban heat island effects while maintaining durability and skid resistance. Using AS in an MMA resin matrix, the formulation effectively lowered pavement surface temperatures while ensuring structural integrity under real-world conditions. Three formulations incorporating MMA resin, a BPO curing agent, silica sand, and one of three thermal fillers (AS, GBs, or PCM) were tested to assess thermal performance and mechanical durability. The following conclusions can be drawn:

1. Optimized material compositions resulted in surface temperature reductions exceeding 10 °C, demonstrating significant heat-reflective performance.
2. Adjusting the resin-to-BPO ratio from 1:0.6 to 1:0.42 reduced curing time to approximately 30 min at 25 °C, without affecting the polymerization kinetics.
3. The AS formulation achieved a maximum compressive strength of 28.2 MPa at 6 wt%, outperforming the PCM and GB formulations, thus proving its superior mechanical performance and workability.
4. The composite material demonstrated stable adhesion, low abrasion rates, and high skid resistance, ensuring long-term safety and durability under dynamic loading.

These findings highlight the potential of integrating hollow ceramic fillers within a polymeric resin matrix to develop thermally adaptive pavement materials. The interplay between material composition, heat mitigation, and durability presents a framework for future research aimed at improving thermal regulation in urban infrastructure. Further investigations into long-term field performance, environmental aging effects, and large-scale implementation will be essential for refining the material’s applications in real-world settings.

## Figures and Tables

**Table 1:** Properties of MMA resin.

Vapor Density (25 °C)	Viscosity (25 °C, mPa·s)	Specific Gravity	Molecular Weight (g/mol)
3.50~6.35	0.53	0.88~0.94	100.10~184.28

**Table 2:** Properties of BPO.

Molecular Formula	Melting Point (°C)	Molecular Weight (g/mol)	Appearance
C_14_H_10_O_4_	104~105	242.23	White powder

**Table 3:** Properties of AS.

Particle Size (µm)	Bulk Specific Gravity (g/cc)	Relative Weight S (g/cc)	PH	Thermal Conductivity (CP)
12~300	0.4	0.6~0.8	6~8	1.53

**Table 4:** Physical properties of GB.

Property	Parameter
Composition	Soda–lime–borosilicate glass
Appearance	White, powdery
Density (g/cc)	0.38
Density Range (µm)	0.30~0.39
Particle Size Range (µm)	40–49
Compressive Strength (MPa)	37.9

**Table 5:** Properties of PCM (*n*-docosane).

Property	Parameter
Molecular Formula	CH_3_-(CH_2_)_20_-CH_3_
Core PCM	n-docosane (PARAFOL 22-95)
Latent Heat	~250 kJ/kg
Phase-Change Temperature	~44 °C
Shell Material	PMMA (Acryl shell)

**Table 6:** Heat resistance test equipment and specifications.

Test Equipment	Specifications
Chamber	180 cm (H) × 50 cm (W) × 50 cm (D)
IR halogen lamp	650 W each
Temperature-measuring instrument (2 pcs.)	Channel: 4 channels; resolution: 0.1 °C

**Table 7:** Summary of results for curing time at different proportions of AS, GB, and PCM.

Content (%)/Materials	Curing Time (min)			
	None	AS	GB	PCM
0	31	-	-	-
5	-	29	30 *	29 *
10	-	30 *	30	30
15	-	30	29	29
20	-	29	30	29
30	-	29	28	30

* Minimum percentage at which stirring became unfeasible (≤150 rpm).

**Table 8:** Temperature differences between reference asphalt specimens and the prototype.

Time (min)	Outdoor (°C)			Indoor (°C)		
	PAC	SMA	DGA	PAC	SMA	DGA
0	0.04	−0.02	−0.02	0.04	0.02	−0.02
30	2.06	2.40	2.50	1.88	2.98	4.08
60	3.10	4.76	4.80	3.06	4.72	5.42
90	3.26	4.96	5.30	3.98	5.94	6.92
120	4.30	6.60	6.88	5.80	7.12	9.32
150	8.98	10.10	10.42	9.42	9.68	9.80
180	10.10	10.36	10.70	10.08	10.76	10.90

**Table 9:** Summary of mechanical performance of developed pavement system.

Test Item		Unit	Test Result	Standard Value
VOC Content		g/L	69.64	≤400
Adhesion strength		MPa	2.12	≥1.5
Abrasion resistance (after 500,000 cycles)	Wear rate	%	0.1	≤1.0
	Skid resistance	BPN	90.2	≥55
Freeze–thaw resistance (outdoor and indoor)		-	No swelling or discoloration	No swelling or discoloration

## Data Availability

The data supporting this study’s findings are available on request from the authors. However, due to confidentiality concerns, the data are not publicly accessible.
